# Prediction of stillbirth low resource setting in Northern Uganda

**DOI:** 10.1186/s12884-022-05198-6

**Published:** 2022-11-19

**Authors:** Silvia Awor, Rosemary Byanyima, Benard Abola, Paul Kiondo, Christopher Garimoi Orach, Jasper Ogwal-Okeng, Dan Kaye, Annettee Nakimuli

**Affiliations:** 1 Department of Obstetrics and Gynecology, Faculty of Medicine Gulu University, Gulu, Uganda; 2 Mulago National Referral Hospital, and Teaching Hospital for Makerere University, P.O.Box 7051, Kampala, Uganda; 3 Department of Mathematics, Faculty of Science, Gulu University, P.O.Box 166, Gulu, Uganda; 4 Department of Obstetrics and Gynaecology, Makerere University, P.O.Box 7062, Kampala, Uganda; 5 Department of Community Health, School of Public Health, College of Health Sciences Makerere University, P.O.Box 7062, Kampala, Uganda; 6 Department of Pharmacology, School of Health Sciences, Lira University, P.O.Box 1035, Lira, Uganda

**Keywords:** Stillbirth, Risk factors, Prediction models, Uganda, Africa

## Abstract

**Background:**

Women of Afro-Caribbean and Asian origin are more at risk of stillbirths. However, there are limited tools built for risk-prediction models for stillbirth within sub-Saharan Africa. Therefore, we examined the predictors for stillbirth in low resource setting in Northern Uganda.

**Methods:**

Prospective cohort study at St. Mary’s hospital Lacor in Northern Uganda. Using Yamane’s 1967 formula for calculating sample size for cohort studies using finite population size, the required sample size was 379 mothers. We doubled the number (to > 758) to cater for loss to follow up, miscarriages, and clients opting out of the study during the follow-up period. Recruited 1,285 pregnant mothers at 16–24 weeks, excluded those with lethal congenital anomalies diagnosed on ultrasound. Their history, physical findings, blood tests and uterine artery Doppler indices were taken, and the mothers were encouraged to continue with routine prenatal care until the time for delivery. While in the delivery ward, they were followed up in labour until delivery by the research team. The primary outcome was stillbirth 24 + weeks with no signs of life. Built models in RStudio. Since the data was imbalanced with low stillbirth rate, used ROSE package to over-sample stillbirths and under-sample live-births to balance the data. We cross-validated the models with the ROSE-derived data using K (10)-fold cross-validation and obtained the area under curve (AUC) with accuracy, sensitivity and specificity.

**Results:**

The incidence of stillbirth was 2.5%. Predictors of stillbirth were history of abortion (aOR = 3.07, 95% CI 1.11—8.05, *p* = 0.0243), bilateral end-diastolic notch (aOR = 3.51, 95% CI 1.13—9.92, *p* = 0.0209), personal history of preeclampsia (aOR = 5.18, 95% CI 0.60—30.66, *p* = 0.0916), and haemoglobin 9.5 – 12.1 g/dL (aOR = 0.33, 95% CI 0.11—0.93, *p* = 0.0375). The models’ AUC was 75.0% with 68.1% accuracy, 69.1% sensitivity and 67.1% specificity.

**Conclusion:**

Risk factors for stillbirth include history of abortion and bilateral end-diastolic notch, while haemoglobin of 9.5—12.1 g/dL is protective.

## Introduction

Stillbirth is the death of a fetus before birth after 20 weeks of gestation [1]. In the early twentieth century, stillbirth was any child who exhibits no sign of life by crying or breathing, or by pulsation in the cord at its attachment to the body of the child, or by beating of the heart and measuring more than 13 inches in length from the top of head to the heel at birth [2]. In the late twentieth century stillbirth was defined as any baby born at 24 weeks of gestation without a sign of life [3]. It can be classified as an early (24 – 27 weeks), late (28 – 36 weeks), or term ( 37 weeks) stillbirth [4].

The global prevalence of stillbirth is approximately 2% [5], with 0.3% occurring in the global north [6, 7], and more than 2% in the global south [5, 8–10]. Women of Afro-Caribbean and Asian origin are more at risk of stillbirths [7, 11–14] and this may be associated with racial disparities in accessing health care [12]. Incidence of stillbirth in Uganda is about 2.0%—3.6% [15, 16]. However, due to challenges in access to care and policies on death registration in the global south, most stillbirths are not registered [17].

When maternal obesity, smoking, chronic hypertension, antiphospholipid syndrome, type 2 diabetes, and insulin requirement are used in a prediction model risk calculator for stillbirth [18], it predicted stillbirths at 60—72% AUC at 75% sensitivity and close to 100% specificity [6, 7, 19]. When maternal history and fetal growth rates were added to maternal history without using the risk calculator, the discriminative performance of the model had a C-statistic of 0.80 [8].

There are limited number of tools built for risk-prediction models for stillbirth within sub-Saharan Africa. With the problems of access to hospital delivery and African ancestry being a risk factor for stillbirth, we set out to develop and validate a prediction model for stillbirth in Northern Uganda.

## Materials and methods

### Study design

A prospective cohort study at St. Mary’s Hospital Lacor, which is one of the teaching hospitals of Gulu University. Using Yamane’s 1967 formula for calculating sample size for cohort studies using finite population size, St. Mary’s hospital Lacor delivers approximately seven thousand mothers per year. Since my study duration was 12 months for recruitment of the mothers, the finite population I could access was about 7,000 mothers. Yamane 1967 formula:

$$\mathrm{Sample size }n =\mathrm{ N }/ 1+\mathrm{Ne}2$$

where N is the finite population size 7,000 mothers.

Margin of error (e) 05%

Therefore *n* = 7,000 / 1 + 7,000(0.05).^2^

*n* = 379.

The required sample size was 379 mothers. We doubled the number (to > 758) to cater for loss to follow-up, miscarriages, and clients opting out of the study during the follow-up period. Recruited 1,285 pregnant mothers 16 – 24 weeks from April 2019 to March 2020. Excluded all with lethal congenital anomalies diagnosed on ultrasound scan especially molar pregnancy, anencephaly, and cystic hygroma. A questionnaire was filled, and uterine artery Doppler sonography was done on all the mothers. The ultrasonography was done by one trained obstetrician. A full foetal anatomical survey was done in addition to the uterine artery Doppler indices (pulsatility and resistive indices, end-diastolic notch). Blood samples were taken for complete blood count, liver and renal function tests, from one thousand (1,000) mothers. The mothers were encouraged to continue with routine antenatal care until the time for delivery. While admitted to the delivery ward, the mothers were followed up by the research team until delivery of the baby. The last mother was delivered at the end of September 2020.

## Outcome

The Apgar score of zero within the first minute of birth at 24 + weeks was taken as stillbirth.

### Statistical analysis

One thousand four (1,004) complete delivery records were obtained. Data were pre-processed using Stata 15.0 and built models using RStudio R version 4.1.1 (2021–08-10). Univariable analysis was done, and all variables with p-values ≤ 0.20 or were known risk factors for stillbirth like age and maternal comorbidities were put together into a logistic regression model. Since the data was imbalanced with few stillbirths, we applied the ROSE technique [20, 21] to create a new dataset by over-sampling stillbirths and under-sampling live births, and obtained a distribution of live births and stillbirth cases as 400 (51.1%) and 383 (48.9%), respectively. The ROSE-derived data set was fitted into a confusion matrix to evaluate the performance of our models (accuracy, sensitivity, specificity) using K-(10)-fold cross-validation. Variables were said to be independent risk factors of stillbirth if their *p*-value < 0.05 in the model. Six models were built.

## Results

**Fig1:**
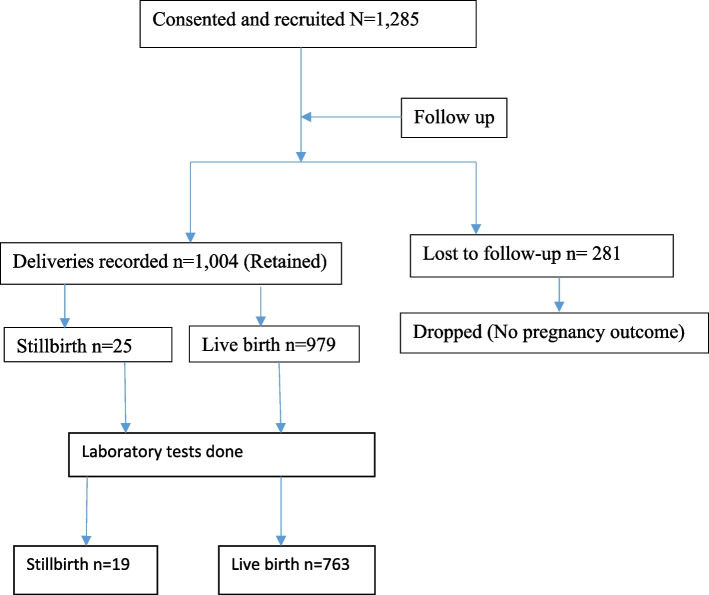
Participant flow through the study

One thousand four (1004) complete delivery records were obtained at the end of the study period.. Of these, seven hundred eighty two mothers had laboratory blood tests done with 2.4% (19) stillbirths and 97.6% (763) live-births. Prevalence of stillbirth was 2.5% (25 out of 1004). There was 979 (97.5%) live births. Seven (28%) out of the 25 deaths occurred intrapartum. Two (8%) of the 25 mothers who lost their babies had a history of previous stillbirth. Two hundred eighty-one mothers were lost to follow-up. Details are found in Fig. 1.

The incidence rates for stillbirth were higher at lower gestation ages, as outlined in Table 1. There were 273 stillbirths per 10^4^ women weeks at < 28 weeks while only 3 stillbirths per 10^4^ women weeks at ≥ 37 weeks.

## Second trimester characteristics of the women who returned to deliver in hospital

Mean maternal age was 26.3 years while 316 participants were first time mothers. Details in Table 2.

Average body-mass index (BMI) was 24.7, and prevalence of multiple pregnancy was 2.4%. Only 0.6% (6) of participants had prenatal hypertension at the time of recruitment. Details in Table 3.

Prevalence of anaemia in pregnancy was high with a mean haemoglogin level of 10.7 g/dL and haematocrit levels of 32.6%. Details in Table 4.

### Unadjusted logistic regression for stillbirth with demographic characteristics

Personal history of preeclampsia and any history of abortion were significantly related to stillbirth while being married or cohabiting was protective. Details in Table 5.

While for the clinical characteristics; systolic hypertension, end diastolic notch, pulsatility and resistive indices were significantly related to stillbirth. Details in table 6.

When laboratory characteristics were used, there were no significant relationship to stillbirth. Details in Table 7.

All the variables with unadjusted p-value of ≤ 0.200 were taken for multivariable analysis to produce the models for prediction of stillbirth. Six models were built in R-studio. The variables are removed from the model in a stepwise manner to remain with the least number of variables with a high AUC. Those variables with p < 0.1 were retained in the model while those with p < 0.05 were taken as independent risk factors for stillbirth.

### Models for prediction of stillbirth

Model 1 examined maternal history and physical examination (details in Table 8). The predictors of stillbirth were parity, age ≥ 35 years, history of abortion and personal history of preeclampsia. Personal history of preeclampsia (aOR = 11.08, 95% CI 1.44—57.34, *p* = 0.0075) and history of abortion (aOR = 2.92, 95% CI 1.07—7.57, *p* = 0.0293) were independent risk factors for stillbirth.

Model 2 examined the uterine artery Doppler indices (details in Table 9). The predictor of stillbirth was presence of end diastolic notch on the uterine artery Doppler flow tracing. Bilateral end diastolic notch (aOR = 4.28, 95% CI 1.54—11.19, *p* = 0.0035) was an independent risk factor for stillbirth.

Model 3 examined the combination of maternal history, physical examination and uterine artery Doppler indices (models 1 and 2) (details in Table 10). The predictors of stillbirth were history of abortion and end-diastolic notch on the uterine artery Doppler flow tracing. The history of abortion (aOR = 3.29, 95% CI 1.24—8.41, *p* = 0.0134) and bilateral end-diastolic notch (aOR = 4.49, 95% CI 1.60—11.88), *p* = 0.0029) were independent risk factors for stillbirth.

Model 4 examined maternal laboratory blood tests (details in Table 11). The predictors of stillbirth were platelet neutrophil ratio, neutrophil count and haemoglobin level. The independent risk factors for stillbirth was platelet neutrophil ratio of > 83.95 (aOR = 5.76, 95% CI 1.12—35.90, *p* = 0.0437). Haemoglobin level of 9.5 – 12.1 g/dL (aOR = 0.32, 95% CI 0.11—0.89, *p* = 0.0287) was protective against stillbirth.

Model 5 examined the combination of maternal history and laboratory tests (models 1 and 4) (details in Table 12). The predictors of stillbirth were history of abortion, parity, age ≥ 35 years and haemoglobin level. The independent risk factors for stillbirth was history of abortion (aOR = 3.10, 95% CI 1.11—8.26), *p* = 0.0254). Haemoglobin level of 9.5 – 12.1 g/dL (aOR = 0.33, 95% CI 0.109—0.95, *p* = 0.0411) was protective against stillbirth.

Model 6 examined the combination of maternal history, physical examination, uterine artery Doppler indices and laboratory tests (models 1, 2 and 4) (details in Table 13).The predictors of stillbirth were personal history of preeclampsia, history of abortion, end-diastolic notch and haemoglobin level. The history of abortion (aOR = 3.07, 95% CI 1.11—8.05, *p* = 0.0243) and bilateral end diastolic notch (aOR = 3.51, 95% CI 1.13—9.92, *p* = 0.0209) were independent risk factors for stillbirth while haemoglobin level of 9.5 – 12.1 g/dL (aOR = 0.33, 95% CI 0.11—0.93, *p* = 0.0375) was protective.

### Evaluation of the models of stillbirth

The models AUC ranges from 66.8% to 75.0%, with accuracies of 63.9% to 68.1%. Details in Table 14.

Model 1 examined maternal history and physical examination (details in Table 8). The predictors of stillbirth were parity, age ≥ 35 years, history of abortion and personal history of preeclampsia. This predicted stillbirth with 65.8% accuracy, 82.4% sensitivity, 48.4% specificity and 71.9% AUC. The details for the models are found in Table 14.

## Discussion

From demographic characteristics of our participants, the predictors of stillbirth were parity, age ≥ 35 years, history of abortion and personal history of preeclampsia. This predicted stillbirth with 65.8% accuracy, 82.4% sensitivity, 48.4% specificity and 71.9% AUC. In Niger state Nigeria, the predictors of stillbirth were maternal comorbidity, rural place of residence, multipara, bleeding during pregnancy, and non-cephalic fetal presentation [8]. Maternal employment was protective of stillbirth [8]. They predicted stillbirth with a C-statistic basic model = 0.80 (95% CI 0.78–0.83), and when ultrasound parameters were added the extended C-statistic model improved slightly to 0.82 (95% CI 0.80–0.83)[8]. In a case–control study in southern Ethiopia, the predictors of stillbirth were women with multiple pregnancy [aOR = 2.98, 95%CI: 1.39–6.36], having preterm birth [aOR = 2.83, 95%CI: 1.58– 508], having cesarean mode of delivery [aOR = 3.19, 95%CI: 1.87–5.44], having no ANC visit [aOR = 4.17, 95%CI: 2.38–7.33], and being hypertensive during pregnancy [aOR = 3.43, 95%CI: 1.93–6.06].[22]. However, these women were recruited after they had given birth. In clinical settings in low resource settings one can use the demographic characteristics above as predictors to identify up to two-thirds of mothers at risk of having stillbirth. Despites the model’s sensitivity of 82.4%, the model’s specificity of 48.4% is low. One will have to put more than twice the number of women identified as at risk of stillbirth in order to get the two thirds of women who will actually get stillbirth.

Combination of uterine artery Doppler indices and maternal history predicted stillbirth by 67.6% accuracy, 75.8% sensitivity and 69.9% AUC. This may be comparable to Akolekar et al.[19] who predicted 55% of all stillbirths, including 75% of those due to impaired placentation and 23% of those that were unexplained or due to other causes, at a false-positive rate of 10% using maternal history and uterine artery Doppler indices. Ultrasound examination is not compulsory in Uganda[23]. It is reserved for a few referral centers, teaching hospitals and private hospitals[24, 25]. Majority of the mothers go through their gestation period without performing a single ultrasound scan.

We predicted stillbirth by 75.0% AUC with 68.1% accuracy, 69.1% sensitivity and 67.1% specificity. This was comparable to the stillbirth-risk calculator [18] validated in Austria at 72% AUC [6]. In the United Kingdom, stillbirth detection rates ranged from 28 to 48% with an AUC of 55.0% to 65.8% even after allowing a 10% false positive rate [7, 19]. In Australia, the detection rate for stillbirth was 45%, with an AUC ranging from 59 to 84% [26]. Similarly, in the United States of America, the detection rate for stillbirth has been 64%—66% AUC [27].

Mastrodima et al. [28] used maternal factors, PAPP-A, Doppler pulsatility index and ductus venosus pulsatility index for veins (DV-PIV), and predicted 40% of all stillbirths and 55% of those due to impaired placentation, at a false-positive rate of 10%. Within the impaired-placentation group, the detection rate of stillbirth < 32 weeks’ gestation was higher than that of stillbirth ≥ 37 weeks (64% vs 42%). This makes the study compare favorably to those conducted in global north. Perhaps the differences seen is due to the differences in the population itself and the technology used for the prediction of stillbirth.

### Research implications

These models may be used in several clinics. Future studies may include a larger number of participants from several locations to validate the models to ensure generalizability.

### Strengths and limitation

This study was a baseline study in Northern Uganda to find out the predictors of stillbirths and to pave way for more research. There was a high number of mothers lost to follow-up.

## Conclusion

In places where ultrasound or laboratory services are not available, the predictors of stillbirths are history of abortion, personal history of preeclampsia, maternal age ≥ 35 years and parity. These variables predict stillbirth by 71.9% AUC with 68.5% accuracy, 82.4% sensitivity and 48.4% specificity.

**Table 1:** Incidence of stillbirth

**Variables**	**Total Population**	**Number of stillbirth**	**% (95% CI)**	**Incidence of stillbirth per 10**^**4**^** women weeks**
No stillbirth	979	0	0%	0
Stillbirth occurred	25	25	2.5% (1.6%—3.7%)	6 (4—9)
Stillbirth occurred < 28 weeks	9	6	66.7% (22.9%—92.5%)	273 (94—379)
Stillbirth ≥ 28—< 37 weeks	119	9	7.6% (3.5%—13.8%)	22 (10—40)
Stillbirth ≥ 37 weeks	876	10	1.1% (0.5%—2.1%)	3 (1—6)

**Table 2:** Social demographic characteristics of the study population at recruitment

**Characteristics (*n* = 1,004)**	**Mean (sd) / Median (IQR) / Proportion (%)**
Maternal age (years) mean (sd)	26.3 (5.5)
Maternal age (years) median (IQR)	26.0 (22.0—30.0)
Single	17 (1.7%)
Married/Cohabiting	987 (98.3%)
Nulliparity	316 (31.5%)
Para 1–2	458 (45.6%)
Para > 2	230 (22.9%)
No history of abortion	810 (80.7%)
Any history of abortion	194 (19.3%)
Umemployed	311 (31.0%)
Informal (casual labourer)	620 (61.8%)
Formal (salaried job)	73 (7.3%)
mean (sd) Gestation age at recruitment (weeks)	20.4 (2.7)
median (IQR) Gestation age at recruitment (weeks)	20.1 (18.6—22.1)
Previous history of preterm birth	85 (12.4%)
No previous history of preterm birth	603 (87.6%)
Personal history of preeclampsia	14 (1.4%)
Not applicable (prime gravida)	316 (31.5%)
No personal history of preeclampsia	674 (67.1%)
Mean (sd) age at menarche (years)	14.4 (1.4)
Median (IQR) age at menarche (years)	14.0 (13.0—15.0)
History of fertility treatment	9 (0.9%)
No history of fertility treatment	995 (99.1%)
Family history of preeclampsia	38 (3.8%)
No family history of preeclampsia	966 (96.2%)
Presence of a chronic illness	90 (9.0%)
No chronic illness	914 (91.0)
Tobacco use in a lifetime	2 (0.2%)
No tobacco use in a lifetime	1,002 (99.8%)
Living with a smoker in one house	104 (10.4%)
No smoker in one house	900 (89.4%)
Alcohol use in pregnancy	56 (5.6%)
No alcohol use in pregnancy	948 (94.4%)

**Table 3:** Clinical characteristics of the study population at recruitment

**Characteristics *n* = 1,004**	**Mean (Sd) / proportion (%)**	**Median (IQR)**
Body mass index	24.7 (3.9)	23.9 (21.8—26.8)
Systolic blood pressure	64.0 (10.4)	63.0 (57.0—70.0)
Diastolic blood pressure	105.7 (12.7)	104.0 (97.0—113.0)
Prenatal hypertension	6 (0.6%)	
No prenatal hypertension	998 (99.4%)	
Singleton pregnancy	980 (97.6%)	
Multiple pregnancy	24 (2.4%)	
No diastolic notch	734 (73.1%)	
Unilateral end diastolic notch	156 (15.5%)	
Bilateral end diastolic notch	114 (11.4%)	
Average Resistive index	0.51 (0.11)	0.50 (0.44—0.58)
Average pulsatility index	0.81 (0.30)	0.75 (0.61—0.96)

**Table 4:** Laboratory characteristics of the population at recruitment

**Characteristics *n* = 787**	**Mean (Sd)**	**Median (IQR)**
Serum ALT	30.4 (27.7)	25.0 (18.0—34.0)
Serum AST	20.1 (23.2)	14.0 (7.0—26.0)
Serum GGT	21.6 (8.5)	20 (15—29)
Serum ALP	153.6 (49.9)	146 (115—179)
Serum bicarbonate	25.4 (2.2)	25 (24—27)
Serum Albumin	4.1 (2.9)	3.9 (3.5—4.1)
Serum Urea	25.3 (26.4)	18 (14—25)
Serum sodium	137.5 (4.0)	137.3 (135.1—139.4)
Serum potassium	4.3 (1.2)	4.2 (3.9—4.5)
Serum chloride	106.3 (4.3)	105.0 (103.5—108.9)
Serum phosphorus	1.3 (0.9)	1.1 (0.9—1.4)
Serum calcium	2.4 (1.2)	2.2 (2.1—2.4)
Serum creatinine	1.0 (0.6)	0.9 (0.8—1.2)
Neutrophil count	3.7 (2.2)	3.5 (2.6—4.6)
Lymphocyte Count	1.8 (0.9)	1.6 (1.3—2.1)
Total White blood cell count	6.3 (2.9)	6.0 (4.9—7.4)
Platelet count	223.9 (69.4)	220 (178—267)
Haemoglobin level	10.7 (2.0)	10.9 (9.5—12.0)
Haematocrit	32.6 (6.7)	33.0 (28.5—33.0)
Mean corpuscular volume	84.3 (7.8)	84.5 (79.9—89.1)
Mean corpuscular haemoglobin concentration	32.9 (2.5)	32.8 (31.4—34.3)

**Table 5:** Unadjusted regression analysis for demographic characteristics for prediction of stillbirth

**Variable**	**OR (95% CI)**	***p*-value**
Maternal age (years) ≥ 35	1.80 (0.63—5.14)	0.271
Married/Cohabiting	0.20 (0.50—0.77)	**0.020**
Nulliparity	1.82 (0.58—5.73)	0.307
Para 1–2	1.38 (0.44—4.29)	0.577
Any history of abortion	2.78 (1.30—6.10)	**0.011**
Informal (casual labourer)	0.67 (0.28—1.57)	0.356
Formal (salaried job)	1.89 (0.60—5.98)	0.277
Previous history of preterm birth	1.09 (0.25—4.76)	0.907
Personal history of preeclampsia	6.15 (1.60—23.62)	**0.008**
age at menarche ≥ 15 years	0.53 (0.16—1.77)	0.305
Family history of preeclampsia	1.06 (0.15—7.63)	0.954
Presence of a chronic illness	0.42 (0.06—3.09)	0.397
Living with a smoker in one house	0.75 (0.18—3.15)	0.697
Alcohol use in pregnancy	1.47 (0.36—6.09)	0.594

**Table 6:** Unadjusted regression analysis for clinical characteristics for prediction of stillbirth

**Variable**	**OR (95% CI)**	***p*-value**
Body mass index > 25 kg/m^2^	0.76 (0.33—1.74)	0.511
Systolic blood pressure ≥ 140 mmHg	5.94 (0.93—38.05)	**0.060**
Diastolic blood pressure ≥ 90 mmHg	1.70 (0.24—12.08)	0.595
Multiple pregnancy	Too few	
Lateral placental location	1.22 (0.29—5.05)	0.788
Unilateral end diastolic notch	1.01 (0.29—3.47)	0.990
Bilateral end diastolic notch	3.68 (1.58—8.58)	**0.003**
Average Resistive index > 0.65 (90th percentile)	3.75 (1.65—8.49)	**0.002**
Average pulsatility index > 1.19 (90th percentile)	3.82 (1.69—8.66)	**0.001**

**Table 7:** Unadjusted regression analysis for laboratory characteristics for prediction of stillbirth

**Variable**	**OR (95% CI)**	***p*-value**
Serum ALT 19—25 IU	2.52 (0.79—8.04)	**0.120**
Serum ALT > 25 IU (> 90th percentile)	0.84 (0.24—2.96)	0.792
Serum AST 4—40 IU (10th—90th percentile)	0.81 (0.19—3.49)	0.782
Serum AST > 40 IU (> 90th percentile)	0.95 (0.14—6.55)	0.756
Serum GGT ≤ 30 IU (Normal lab range)	1.34 (0.39—4.54)	0.639
Serum ALP ≤ 98 IU (low lab range)	1.44 (0.20—10.45)	0.717
Serum bicarbonate 24—27 (25th—75th percentile)	4.10 (0.54—30.93)	**0.172**
Serum bicarbonate > 27 (> 75th percentile)	3.77 (0.43—33.36)	0.233
Serum albumin 3.5—4.1 g/dL	0.46 (0.15—1.42)	**0.180**
Serum Albumin < 3.5 g/dL	1.27 (0.43—3.70)	0.667
Serum urea 14—25 mg/dL (25th—75th percentile)	1.50 (0.42—5.40)	0.534
Serum urea > 25 mg/dL (> 75th percentile)	1.85 (0.0.47—7.30)	0.379
serum creatinine 0.61—1.50 mg/dL (10th—90th percentile)	0.62 (0.21—1.86)	0.395
Serum creatinine > 1.50 mg/dL	0.35 (0.40—3.07)	0.343
Neutrophil count 2.63—4.54 cells/microlitre	0.92 (0.31—2.71)	0.881
Neutrophil count > 4.54 cells/microlitre	1.00 (0.29—3.40)	1.000
Lymphocyte Count 0.9—3.9 cells/microlitre	0.33 (0.10—1.12)	**0.075**
Lymphocyte Count > 3.9 cells/microlitre	1.89 (0.34—10.42)	0.465
Total White blood cell count 4000–11,000 cells / microlitre	1.10 (0.26—4.70)	0.900
Total White blood cell count > 11,000 cells / microlitre	2.91 (0.28—30.25)	0.372
platelet count 178—266 cells / microliter (25th—75th percentile)	1.60 (0.45—5.76)	0.470
Platelet count > 266 cells / microliter (> 75th percentile)	1.93 (0.49—7.60)	0.348
Haemoglobin level < 9.5 g/dL (< 25th percentile)	2.78 (0.76—10.12)	**0.120**
Haemoglobin level 9.5—12.1 g/dL (25th—75th percentile)	1.02 (0.27—3.89)	0.981
Haematocrit 30—39.9% (25th—75th percentile)	0.50 (0.20—1.25)	**0.140**
Haematocrit ≥ 40% (> 75th percentile)	0.49 (0.06—3.83)	0.501
Mean corpuscular volume 79.9—89.2 fl (25th—75th percentile)	1.30 (0.42—4.04)	0.647
Mean corpuscular volume < 79.9 fl (< 25th percentile	0.97 (0.25—3.82)	0.965
Mean corpuscular haemoglobin concentration 31.5—34.4 g/dL	1.05 (0.40—2.75)	0.923
Mean corpuscular haemoglobin concentration < 31.5 g/dL	0.18 (0.22—1.47)	**0.110**

**Table 8:** Model 1 using maternal history for prediction of stillbirth

**Variable**	**OR (95% CI)**	***p*-value**
Personal history of preeclampsia	11.08 (1.44—57.34)	**0.0075**
History of abortion	2.92 (1.07—7.57)	**0.0293**
Age ≥ 35 years	4.29 (0.72—20.72)	0.0851
nullipara	5.37 (1.10—36.24)	0.0576
para 1—2	2.28 (0.48—13.67)	0.3284
Intercept	0.005 (0.001—0.02)	0.0000

**Table 9:** Model 2 using uterine artery Doppler indices for prediction of stillbirth

**Variable**	**OR (95% CI)**	***p*-value**
Unilateral	0.40 (0.02—2.09)	0.3843
Bilateral	4.28 (1.54—11.19)	**0.0035**
Intercept	0.02 (0.01—0.03)	0.0000

**Table 10:** Model 3 using combination of maternal history and uterine artery Doppler indices for prediction of stillbirth

**Variable**	**OR (95% CI)**	***p*-value**
History of abortion	3.29 (1.24—8.41)	**0.0134**
Unilateral	0.38 (0.02—2.01)	0.3618
Bilateral	4.49 (1.60—11.88)	**0.0029**
Intercept	0.01 (0.006—0.03)	0.0000

**Table 11:** Model 4 using maternal laboratory tests for prediction of stillbirth

**Variable**	**OR (95% CI)**	***p*-value**
Platelet neutrophil ratio of 47.04—83.95	1.80 (0.46—9.00)	0.4232
Platelet neutrophil ratio of > 83.95	5.76 (1.12—35.90)	**0.0437**
Neutrophil count of (2.63—4.54) *1000	2.14 (0.60—8.12)	0.2453
Neutrophil count of (> 4.54) *1000	4.16 (0.77—22.81)	0.0958
Haemoglobin level of 9.5—12.1 g/dL	0.32 (0.11—0.89)	**0.0287**
Haemoglobin level of > 12.1 g/dL	0.33 (0.07—1.14)	0.1027
Intercept	0.01 (0.001—0.06)	0.0000

**Table 12:** Model 5 using combination of maternal history and laboratory tests for prediction of stillbirth

**Variable**	**OR (95% CI)**	***p*-value**
History of abortion	3.10 (1.11—8.26)	**0.0254**
Age ≥ 35 years	4.87 (0.79—24.57)	0.0677
nullipara	5.09 (1.02—35.71)	0.0715
para 1—2	2.51 (0.52—15.59)	0.2831
Haemoglobin level of 9.5—12.1 g/dL	0.33 (0.109—0.95)	**0.0411**
Haemoglobin level of > 12.1 g/dL	0.27 (0.06—0.99)	0.0656
Intercept	0.01 (0.001—0.005)	0.0000

**Table 13:** Model 6: Combination of maternal history, uterine artery Doppler indices and laboratory tests for prediction of stillbirth

**Variable**	**OR (95% CI)**	***p*-value**
Personal history of preeclampsia	5.18 (0.60—30.66)	0.0916
History of abortion	3.07 (1.11—8.05)	**0.0243**
Unilateral	0.37 (0.02—1.98)	0.3507
Bilateral	3.51 (1.13—9.92)	**0.0209**
Haemoglobin level 9.5—12.1 g/dL	0.33 (0.11—0.93)	**0.0375**
Haemoglobin level > 12.1 g/dL	0.30 (0.06—1.07)	0.0850
Intercept	0.03 (0.01—0.07)	0.0000

**Table 14:** Evaluation of the models for stillbirth

**Model**	**Accuracy**	**Sensitivity**	**Specificity**	**AUC**
Model 1 (Maternal history and exam)	65.8	82.4	48.4	71.9
Model 2 (Uterine artery Doppler indices)	63.9	88.7	37.9	66.8
Model 3 (History and uterine artery Doppler indices)	67.6	75.9	59.0	69.9
Model 4 (lab tests)	65.3	71.6	58.7	69.7
Model 5: (combination of history and laboratory tests)	68.0	67.1	69.0	74.4
Model 6: (combination of maternal history, Doppler indices and laboratory tests)	68.1	69.1	67.1	75.0

## Data Availability

The corresponding author Dr. Silvia Awor or Makerere University Directorate of Research and Graduate training can be contacted to request for the data and other materials.
